# Foot Pressure Wearable Sensors for Freezing of Gait Detection in Parkinson’s Disease

**DOI:** 10.3390/s21010128

**Published:** 2020-12-28

**Authors:** Andrea Marcante, Roberto Di Marco, Giovanni Gentile, Clelia Pellicano, Francesca Assogna, Francesco Ernesto Pontieri, Gianfranco Spalletta, Lucia Macchiusi, Dimitris Gatsios, Alexandros Giannakis, Maria Chondrogiorgi, Spyridon Konitsiotis, Dimitrios I. Fotiadis, Angelo Antonini

**Affiliations:** 1 UOC Recupero e Riabilitazione Funzionale, Ospedale di Lonigo, Azienda ULSS 8 Berica, 36045 Lonigo, Italy; andrea.marcante@aulss8.veneto.it; 2 Department of Neuroscience, University of Padova, 35128 Padova, Italy; angelo.antonini@unipd.it; 3 Fondazione Ospedale San Camillo IRCCS, 30126 Venezia, Italy; 4 Laboratory of Neuropsychiatry, IRCCS Santa Lucia Foundation, 00179 Rome, Italy; c.pellicano@hsantalucia.it (C.P.); f.assogna@hsantalucia.it (F.A.); francesco.pontieri@uniroma1.it (F.E.P.); g.spalletta@hsantalucia.it (G.S.); l.macchiusi@hsantalucia.it (L.M.); 5 Department of Neurology, Belcolle Hospital, 01100 Viterbo, Italy; 6 Department of Neuroscience, Mental Health and Sensory Organs (NESMOS), Sapienza University of Rome, 00185 Rome, Italy; 7 Department of Materials Science, Unit of Medical Technology and Intelligent Information Systems, University of Ioannina, Stavrou Niarchou Av., University Campus, 451 10 Ioannina, Greece; d.gatsios@uoi.gr (D.G.); fotiadis@uoi.gr (D.I.F.); 8 Department of Neurology, Medical School, University of Ioannina, Stavrou Niarchou Av., University Campus, 451 10 Ioannina, Greece; papadates@gmail.com (A.G.); mchond@cc.uoi.gr (M.C.); skonitso@uoi.gr (S.K.)

**Keywords:** Parkinson’s disease, freezing of gait, wearable device, insoles, accelerometer, gait monitoring

## Abstract

Freezing of Gait (FoG) is a common symptom in Parkinson’s Disease (PD) occurring with significant variability and severity and is associated with increased risk of falls. FoG detection in everyday life is not trivial, particularly in patients manifesting the symptom only in specific conditions. Various wearable devices have been proposed to detect PD symptoms, primarily based on inertial sensors. We here report the results of the validation of a novel system based on a pair of pressure insoles equipped with a 3D accelerometer to detect FoG episodes. Twenty PD patients attended a motor assessment protocol organized into eight multiple video recorded sessions, both in clinical and ecological settings and both in the ON and OFF state. We compared the FoG episodes detected using the processed data gathered from the insoles with those tagged by a clinician on video recordings. The algorithm correctly detected 90% of the episodes. The false positive rate was 6% and the false negative rate 4%. The algorithm reliably detects freezing of gait in clinical settings while performing ecological tasks. This result is promising for freezing of gait detection in everyday life via wearable instrumented insoles that can be integrated into a more complex system for comprehensive motor symptom monitoring in PD.

## 1. Introduction

Parkinson’s Disease (PD) is the second most common neurodegenerative disorder, and its prevalence is increasing due to the population aging [1,2]. The efficacy of pharmacological therapy progressively reduces along the disease course, leading to motor and non-motor fluctuations. Medical visits typically occur with a several week interval, limiting the possibility to capture Freezing of Gait (FoG). FoG is defined as a sudden and brief episode of the inability to produce effective forward stepping and can appear at the initiation of gait (patients report feeling their feet “glued to the floor” while their upper body continues its original trajectory), while turning, or passing through narrow spaces (e.g., through a door) [3]. Unfortunately, as the disease progresses, FoG becomes less sensitive to drug therapies, and despite their careful management, advanced treatment (i.e., deep brain stimulation, Duodopa™), and personalized rehabilitation therapies, people experiencing FoG are often exposed to falls and fall-related injuries [4,5,6]. Being sudden, brief, and frequently linked to the OFF phase, FoG episodes do not necessarily and spontaneously occur during neurological examinations in scheduled visits. Therefore, it is difficult to correctly evaluate FoG severity and impact on daily life activities.

Many technological solutions based on wearable devices have been recently developed and tested with the aim of finding algorithms that could automatically and objectively detect gait disturbances [7,8,9,10,11], with particular interest in FoG [12]. Compact size, ease of use, and affordable cost make these systems a potential aid for clinicians to monitor patients’ symptoms in natural environments and to obtain an objective evaluation of clinical status [13,14]. According to Rovini et al. [15], tremor, motion analysis (e.g., via the monitored Timed Up and Go test (TUG), FoG, postural instability), and fluctuation analysis (evaluation of the ON and OFF conditions) are the most studied features of PD. FoG detection has been mainly performed using acceleration and angular velocity-based techniques from body-worn sensors. More specifically, Moore et al. [16] found that FoG events are not described by purely stochastic events, but are indeed well defined in the low frequency band (3–8 Hz) and proposed an index to objectively identify FoG events offline. Building on this finding, algorithms manipulating acceleration signals gathered from shin-mounted sensors have been introduced [17,18] and applied to study the occurrence of FoG while turning [19]. A further development of this approach called for the use of both accelerometer and gyroscope signals collected from sensors mounted on the lateral aspect of tibia and segmented on a gait cycle basis. Segmented signals were used to feed an algorithm based on Pearson’s correlation coefficient to: (i) separate epochs of “normal” strides from those that differ significantly from the so-called “representative” stride; and (ii) classify the latter group based on the frequency content of the acceleration signals and on the spatio-temporal parameters of gait estimated fusing accelerometers and gyroscopes measures [20,21]. This approach was only validated for people with PD displaying a dominantly normal gait pattern.

All the reported approaches are applicable to identify FoG episodes while walking and could then fail when a lack of or small movements occur. Indeed, different subtypes and features of FoG can be observed [20,21]: e.g., FoG with legs trembling and FoG with complete motor blocks in absence of effective steps. In this perspective, Center Of Pressure (COP) trajectories could be informative about the intention of movement [21,22], especially when looking at the frequency domain. Thus, although being moderately used and validated, pressure sensors are a promising alternative to inertial sensors in FoG detection. The aim of this study was to present and test an innovative, unobtrusive, and wearable system to be used to monitor patients’ activity and detect FoG episodes in an ecological environment. This device will analyze foot pressure distribution changes during gait, complemented with acceleration data. This approach could become part of a home-based test scheduled by clinicians and a monitoring system for PD patients in everyday life.

## 2. Materials and Methods

### 2.1. Participants

A total of 20 patients (14 males and 6 female; see Table 1 for complete demographic data) were recruited and gave informed consent to participate in the study, specifically for data and video recording. Ethical approval was granted by the competent local authorities, and the study was performed in accordance with the standards of the Declaration of Helsinki. Ten hospitalized patients were recruited at IRCCS Fondazione Ospedale San Camillo (Venice, Italy), 5 outpatients at IRCCS Santa Lucia (Rome, Italy), and 5 outpatients at the University Hospital of Ioannina (Ioannina, Greece). They were all diagnosed with idiopathic Parkinson’s disease according to the UK Brain Bank criteria [23] and had motor fluctuations (Hohen and Yahr score ≥ 3 in the OFF state [24]). Patients with severe cognitive impairment, assessed through the Montreal Cognitive Assessment Test [25], and with other neurological comorbidities (such as a history of stroke) were excluded. Six out of 20 patients had dyskinesia, whereas the remaining participants showed bradykinesia and tremor. Only three patients self-reported frequent falls, and eight of them reported rare episodes. There was one patient treated with Duodopa, and three of them had Subthalamic Nucleus Deep Brain Stimulation (STN-DBS). These reported differences among patients did not affect the obtained results, as the proposed methodology aimed to detect FoG episodes that occurred independently of the treatment.

### 2.2. Data Collection

The Sensor Insoles (SI, Version 1) produced by Moticon GmbH (Munich, Germany) consisted of 13 capacitive pressure sensors, a 3D accelerometer, rechargeable batteries, a built-in recording data memory, and a Bluetooth device. The SI allowed recording (sampling rate 50 Hz): (i) foot pressure distribution, (ii) foot accelerations, and (iii) other computed measures (in particular, total force and COP sequences). Data were stored on the on-board memory and then downloaded to a PC via OpenGo (Moticon GmbH, Munich, Germany).

Each patient underwent an 8 session recording protocol (4 ON state sessions and 4 OFF state sessions). The OFF-sessions were performed in the morning, before the first intake of L-Dopa or dopamine agonist or before starting Duodopa infusion. The ON sessions were scheduled in the 45–90 min after the intake of the usual daily morning dose (administered subsequently to the OFF sessions). Before each session, a clinical evaluation based on selected UPDRS (Unified Parkinson’s Disease Rating Scale) III sub-items was performed [28]. For each recording session, patients were asked to place the insoles in their own shoes and wear them. Prior to the experiments, a zero-level calibration for the pressure was performed via the OpenGo software prior to starting recording, getting rid of the offset due to the foot-insole-shoe interaction.

Table 2 shows the recorded motor tasks of the protocol. Being part of a larger project, the tasks listed in Table 2 were needed to simulate daily activities with the aim of triggering specific disease symptoms, with particular attention to FoG. Among these tasks, the 2 Minute Walking Test (2MWT), the 360° turn test, and Timed Up and Go (TUG) were included as the benchmark for FoG triggering and evaluation [19,29,30]. The FoG detection algorithm was then applied to those tasks including walking: 3, 4b-c-d-e, 5 in Table 2. Each session of data collection consisted of all the tasks listed, which were performed always in the same order. The duration of each session was heavily influenced by the motor state of the participants: one with mild symptoms in the ON state could easily complete the single session in 10–15 min, but sessions could also last 30–45 min. One minute of rest was administered between tasks, and extra time was admitted if the participants asked for it.

**Figure 1:**
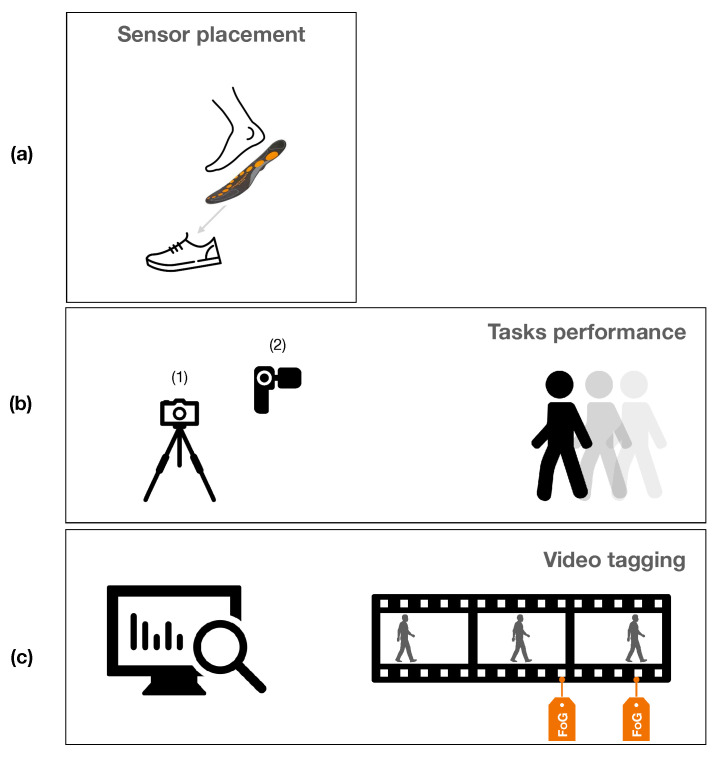
Data collection and processing protocol: (**a**) placement of the sensor insoles in the participants’ shoes, then worn; (**b**) video recording of task performance (open a door, getting up from the bed, walking, etc.); (**c**) video tagging of Freezing of Gait (FoG) episodes (onset, duration, end).

All performed tasks were recorded using two cameras. One camera was placed on a tripod to allow recording of movements on the frontal plane of the participants for Tasks 3, 4a, 4f, and 5 in Table 2. An additional camera was used by a research assistant to follow the participants during the performance of the ecological tasks. Raw data coming from the SI were synchronized with video recordings using the OpenGo software (Figure 1).

### 2.3. FoG Detection Algorithm

An algorithm was implemented and copyrighted within OpenGo to automatically detect a wide range of FoG subtypes, accounting for pressures, forces, and accelerations (copyright of Moticon GmbH, Munich, Germany). Before looking at the data, the main features of the walking of PD patients were extensively discussed with an expert neurologist (A.A.). Particular attention was payed to FoG. A set of possibly relevant quantities to investigate was identified building on this discussion and then looking at data gathered from FoG episodes, differentiating also from non-FoG signals. In particular:

1. Looking at the total contact when no actual step is performed, but rather FoG with trembling of legs occurs leads to a weight shift between the left and right sides, not necessarily in combination with feet leaving the ground, but with a frequency that is higher than during normal walking;
2. A gait cycle normally lasts about 1 s, and two peaks of the total contact force should be observed within this time span. Singular peaks of the weight curve (i.e., the total force) are in contrast to normal walking curves;
3. Motor blocks could lead to a moderate excursion of the COP in the anterior-posterior direction at relatively high frequency (i.e., participants trying to step forward, but not even starting the step);
4. A low range of the acceleration in the vertical axis indicates that feet are actually not being lifted.

To numerically define the above indicators, the following features were computed after segmenting the collected signals in windows of 1 s:

- fraction of the weight span (*w*), defined as the maximum minus the minimum values of the total force under a foot relative to the estimated body weight (defined as 90% of the body weight measured during the clinical assessment);
- dominant frequency of the total force curve as obtained from the Fourier transform of the signal ($f_{TF}$);
- dominant frequency of the COP curve (anterior-posterior component) as obtained from the Fourier transform of the signal ($f_{COP}$);
- range of the acceleration curve (vertical axis, $r_{acc}$).

The dominant frequency (both for $f_{TF}$ and $f_{COP}$) was computed using the FFT command of the NumPy library. It is defined as the frequency of the maximum peak of the signal spectrum neglecting the DC component. The strength of this approach is that FoG episodes can be evaluated with a temporal resolution of one second.

A criterion function was defined as the product of the above features:

(1) $$f_{crit}=\left\{\begin{array}{cc} f_{TF}\cdot f_{COP}\cdot \left|4-r_{acc}\right| & \mathrm{when}\;w\geq 0.7\\ 0 & \mathrm{when}\;w<0.7 \end{array}\right.$$

Setting $f_{crit}$ to 0 if w<0.7 effectively limits the criterion function to consider walking sequences only. The term $|4-r_{acc}|$ indicates that high weight is given to those events displaying low values of acceleration (i.e., the lower the $r_{acc}$, the higher $|4-r_{acc}|$), which are likely associated with FoG episodes. The choice of 4 as the upper bound was data-driven: accelerations never reached ranges of values greater than 3.5g.

Using automated parameter variation with the annotated FoG duration being the target value, a good balance to minimize false positives and false negatives was obtained choosing a threshold of 12.0 for $f_{crit}$. Any time window where $f_{crit}$ exceeded 12.0 was considered a window with FoG.

### 2.4. Data Analysis

The FoG episodes automatically detected via the above described algorithm are hereinafter addressed as AD-FoG and their duration as AD-FoGt. Blinded from the automatic results, an expert clinician evaluated the video recordings and marked the start time and stop time of each FoG episode (AN-FoG, i.e., annotated FoG episodes). The relevant duration of each AN-FoG episode was then estimated (AN-FoGt). The algorithm was developed to match the overall duration of FoG episodes during the recordings and not to exactly match the annotated start/stop of FoG for three main reasons: (i) the aim of our algorithm was not to exactly match the onset and end of FoG episodes, but rather to provide a binary answer (yes/no) to the question “Did the patient experience FoG ?”; (ii) onset and end time points sometimes cannot be unambiguously defined even by a very well-trained observer; and (iii) FoG episodes could occur in video blinded areas. Indeed, a research assistant was instructed to follow the participants and hold a video camera to film the task performance, but he was also instructed to prioritize the performance in the most possible ecological manner, and thus to not impede participants natural movement. This led to the loss of some FoG episodes’ recording, however not exceeding 5% of the total amount. Each detected episode contributed to the overall duration, but detected false positives could significantly affect the algorithm’s performance. AD-FoG episodes were then tested against the AN-FoG via a Bland–Altman plot [31] to validate the algorithm’s ability to detect the overall duration of FoG episodes within a monitoring time window. The *i*-th AD-FoGt that did not exceed a threshold set at 0.7% of the overall FoG duration was filtered out and considered as a short false positive. The threshold value (0.7%) was chosen as the optimal value a posteriori, acknowledging the best matching false positive/false negative of the full dataset (i.e., from all the participants).

IBM SPSS Statistics v23.0 was used to perform descriptive (mean and standard deviation values) and inferential statistics to verify that the clinical outcome differences were consistent with the ON state and induced OFF state sessions. A repeated measures ANOVA (*p*-value = 0.05) was used to test each UPDRS sub-item and each output of the clinical tests. The chi-squared was instead used to test the presence/absence of dyskinesia between the ON and OFF conditions.

### 2.5. Wearability

Participants were asked to fill in a questionnaire regarding the perceived safety, acceptance, and comfort of the tested system at the end of the 8 sessions. Participants were asked to answer questions using a combination of scales [32], which included BORG CR-10 to evaluate perception and experience (including pain and exertion) and the Comfort Rating Scale (a 21 point scale) to evaluate the wearability and acceptance of the device. These scales have been already applied to Parkinson’s disease [33,34].

## 3. Results

A total of 140 out of 160 (expected) sessions were recorded. Drop-outs were due to excess of fatigue experienced (four patients, 12 sessions) or the presence of severe motor symptoms (three patients, eight sessions). As expected, UPDRS scores and motor tests (e.g., 2MWT, TUG, etc.) performed in the ON and OFF state were found to be significantly different (the highest *p*-value was 0.027 obtained for the UPDRS finger tapping), worsening from the ON to OFF state evaluations (see Table 3). The only exception was obtained for the UPDRS Item 3.17d “Tremor Severity Left Leg”: OFF score of 0.36±0.84.

### 3.1. FoG Detection

**Figure 2:**
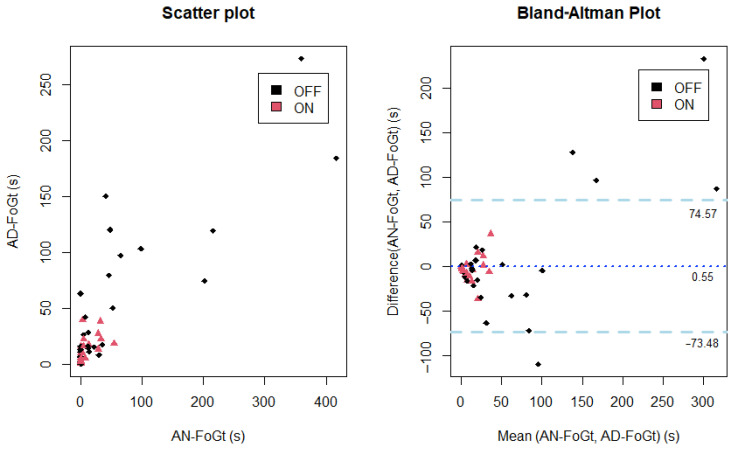
Scatter plot of the clinician’s annotated episodes (AN-FoGt) and algorithm detected FoG episodes’ duration (AD-FoGt) on the left side and Bland–Altman plot on the right.

Among the total number of sessions recorded, fifty-three out of 140 (38%) contained AN-FoG episodes. The AN-FoGt lasted between zero and 417 s, with an average of 33 s. In the OFF state, the average AN-FoGt was 61 s, compared to 7 s in the ON state. In the case of large AN-FoGt, the algorithm generally underestimated the AD-FoGt, but the Bland–Altman plot (Figure 2, right side) shows a good agreement between the AN-FoGt and AD-FoGt. FoG episodes that respected the threshold (0.7%) were classified as true episodes. As a result, ninety percent of the measurements were correctly detected as either being FoG or non-FoG episodes after applying the threshold of 0.7% of the overall FoG duration within the testing time window. The false positive rate was 6% (specificity 94%) and the false negative rate 4% (sensitivity 96%).

### 3.2. Wearability

**Figure 3:**
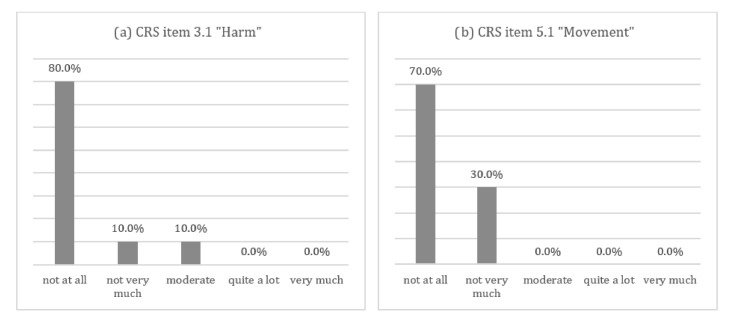
Chart of the Comfort Rating Scale’s (CRS) most representative items: (**a**) patients’ answer to the Comfort Rating Scale Item 3.1 “Harm”: the device caused me some harm (e.g., headache, pain, itching, irritation, etc.); (**b**) patients’ answer to the Comfort Rating Scale Item 5.1 “Movement”: the devices badly affects the way I move or slows down or obstructs my movements.

The results of the wearability assessment showed a good acceptance of the sensor insoles. Figure 3a,b show that the majority of patients did not perceive the devices as interfering with their movements or causing harm. All subjects reported no complaints according to the BORG CR-10 scale, except for one out of 20 participants reporting a “weak” complaint on the foot plant. Comfort Rating Scale (CRS) assessment reported a low median value for each subscale (median; min-max): emotion (0; 0–5.5), attachment (1; 0–6), harm (0; 0–6), perceived change (0; 0–5), movement (0; 0–5), and anxiety (0; 0–3), with an overall score corresponding to “The system is wearable” [32]. Only four out of 20 patients (20%) reported minor complaints related to increased sweating due to reduced perspiration caused by the device material.

## 4. Discussion

The aim of this study was to test a wearable system to automatically detect FoG episodes in PD patients, combining the analysis of foot pressure distributions and signals from a 3D accelerometer to be used in everyday life to detect the presence or absence of FoG. Different from the approaches based only on accelerometer measures [16,17,18], our approach is able to detect different kinds of FoG episodes. The analysis of the pressure distributions and pressure-related parameters under feet measured by the sensor insoles can indeed differentiate between FoG episodes happening at step initiation or with complete motor blocks (i.e., the absence of effective steps), which the accelerometer-based algorithm failed to recognize. To the best of our knowledge, only one other study tested the use of pressure sensors to detect FoG episodes in PD [20]. Even if they used a different approach in data analysis and not a 3D accelerometer to complement the pressure data, the authors demonstrated that their system was sensitive to various freezing with results obtained from only 24 FoG episodes. Thus, the present study is the first testing a large pressure- and acceleration-based system in a large dataset (140 episodes from 20 patients). As in other studies, we tested the device in a safe and supervised clinical environment, but the motor assessment protocol was designed with structured and complex sessions consisting of clinical tests (i.e., Timed Up and Go) and ecological tasks reflecting daily life situations. Additionally, to increase the variability of measurements, we repeated the data collection four times during both the ON and OFF state.

The scores for the UPDRS sub-items assessing bradykinesia were higher than those assessing tremor, reflecting a prevalent bradykinetic phenotype for the enrolled participants. 2MWT and TUG results in the ON state were consistent with those obtained for a population of moderate-advanced PD [35,36]. Time to complete TUG in the OFF state were significantly higher than in the ON state. However, it is worth noting that two patients were barely able to complete the test, with a consequent increase time to complete it. The results of the 360° turn test in the ON state were also descriptive for a population with a Hohen–Yahr score between two and three [36]. We observed a higher value for turning time in the OFF state, especially for left rotation: this was mainly related to FoG that can be evoked during this kind of test.

For this study’s aim, the results highlighted a good agreement between the FoG episodes annotated by an expert clinician and those automatically detected by the tested algorithm. Although underestimating long lasting FoG episodes, the algorithm reached high sensitivity and specificity (96% and 94%, respectively). The major disagreement was also associated with episodes in the OFF sessions that were artificially caused by the total absence of drugs, which is very unlikely in natural situations. This shows that our new approach is promising for both ambulatory and other applications to provide clinicians with a binary answer (yes/no) to the question “Did the patient experience FoG?”. Our algorithm performances are in line with other proposed devices, whose sensitivity and specificity range from 67% up to 100% [12]. We acknowledge that further investigations are worth undertaking on a larger number of participants with PD, also exploring the application of our approach beyond hospital walls and movement analysis laboratories. Future works are surely worth performing to explore also different parameters and different combinations of them to build new criterion functions, possibly achieving a further improvement of the presented results.

All recorded signals were observed and analyzed to understand their pattern during the FoG episodes, and their difference from non-FoG signals. Although the intermediate steps of the data analysis are copyrighted, an effective combination of features in distinguishing FoG episodes was: (a) weight shifts between the left and right side, not necessarily in combination with the feet leaving the ground, but with a frequency higher than during normal walking; (b) singular peaks of the weight curve (i.e., the total force applied on the foot), which is in contrast to normal walking, where typically, two peaks occur; (c) a moderate excursion of the COP in the anterior-posterior direction at relatively high frequency; (d) a low range of values of the acceleration along the vertical axis. Being also based on COP measurement, the novel system detects FoG episodes also characterized by trembling without effective steps, for example occurring at gait initiation, overcoming the limitations observed when accelerometers are used [20,21]. Finally, insights and information coming from continuous and specific sensor-based monitoring of these features over time combined with patient reported outcome and symptoms’ clinical assessment, may support clinical decisions in managing PD symptoms [37].

Consistent with recent findings [38], the overall feasibility and high acceptance of the insole-based pressure sensor for walking and FoG assessment should be highlighted. They should be considered as an effective tool in telemedicine and suitable for home-based clinical assessment with simple tests used to assess patients’ walking and freezing. Feedback provided by patients leads to the conclusion that the acceptance and ease of use of the sensor insoles are goals that have already been achieved, although they suggest that high transpiration materials should be taken into account to reduce the burden of PD hyperhidrosis and sweating dysfunction.

## 5. Conclusions

Freezing of gait assessment is particularly important in Parkinson’s disease, especially at the late stage. Healthcare processes move towards the Health Technology Assessment (HTA) framework, aiming to extend evaluations beyond hospital walls. The tested system is an integration of well-known accelerometer-based devices combined with a foot pressure sensor and the parameters of the pressure distribution. Our results demonstrate a high degree of agreement with clinical evaluation in a supervised environment enabled by a ecological motor task as a reliable tool for future application in home-based contexts (unsupervised) for everyday use, paving the way for this system to be: (i) used in daily life to evaluate freezing of gait; and (ii) integrated with other wearables for comprehensive motor symptom monitoring in PD.

## Figures and Tables

**Table 1:** Main demographic data: age, education, disease duration, Dopamine Agonists Equivalent Daily Dose (DAED; dopamine agonists were administered to only 12 participants out of 20), and Levodopa Equivalent Daily Dose (LEDD) according to [26]. The raw score of the Montreal Cognitive Assessment was then corrected according to the Italian normative data for cognitive impairment staging [27].

Demographic and Clinical Data	Notes	Mean	SD	Min	Max
Age (years)	–	68.60	10.69	44	88
Education (years)	–	8.00	5.04	2	18
Disease duration (years)	–	10.45	5.35	3	26
DAED	received by only 12 participants	220.25	131.33	80	550
LEDD	–	1008.87	624.41	133	2860.5
MoCA	–	22.06	5.627	12	30

**Table 2:** List of motor tasks executed during each recording session.

Motor Recording Protocol		
**1.**	Lie on the bed for 1 min	
**2.**	Rise from the bed, and sit on a chair located beside the bed for 1 min	
**3.**	Perform the Timed Up and Go test (TUG)	
**4.**	Stand up from the chair and perform a series of activities:	
	**a.**	Stand (without moving) for 1 min (While standing, avoid communication with the patient. The clinician stands in front of the patient, not on the patient’s side, to avoid the patient turning towards the clinician.). The camera is placed in front of the patient, in order to capture postural abnormalities on the frontal plane
	**b.**	Walk for a distance of 5 m, and open the door (with the arm with the wristband); then walk through the door, and exit the room; go back in the room, and close the door (repeat 3 times; this should evoke freezing, if present).
	**c.**	Two minute walking test
	**d.**	Walk back to the room
	**e.**	Stop, and drink a few sips from a glass of water (repeat the sequence: take the glass with the arm with the smartband; drink and leave the glass 3 times)
	**f.**	Stand (without moving) for 1 min. While standing, the clinician avoids communication with the patient. The clinician stands in front of the patient, not on the patient’s side, to avoid the patient turning towards the clinician. This time, the patient is recorded from one side (the side recorded is the one where the patient is wearing the wristband), in order to capture any postural abnormalities for the sagittal plane;
**5.**	360° turn test clockwise + anticlockwise (record seconds and number of steps)	

**Table 3:** ON-OFF state comparison within subjects for the main clinical scales and sub-item (UPDRS) and tests: 360° turn test (360° turn, time, and steps), Timed Up and Go (TUG), 2 Minute Walk Test (2MWT).

Variable	OFF State		ON State		Statistics		
	*M*	*SD*	*M*	*SD*	*df*	*F*	*p*
UPDRS Item 3.3b: Rigidity severity R	1.20	0.88	0.65	0.61	1	17.5	0.000
UPDRS Item 3.3c: Rigidity severity L	1.29	0.91	0.71	0.82	1	15.015	0.000
UDPRS Item 3.4a: Finger tapping R hand	1.55	0.90	1.20	0.83	1	5.29	0.023
UDPRS Item 3.4b: Finger tapping L hand	1.82	0.86	1.49	0.83	1	4.998	0.027
UDPRS Item 3.10: Gait	1.73	0.87	1.12	0.74	1	19.46	0.000
UDPRS Item 3.17a: Tremor severity R arm	0.45	0.85	0.19	0.43	1	5.394	0.022
UDPRS Item 3.17b: Tremor severity L arm	0.65	0.87	0.20	0.58	1	12.516	0.001
UDPRS Item 3.17c: Tremor severity R leg	0.30	0.70	0.07	0.26	1	6.521	0.012
UDPRS Item 3.17d: Tremor severity L leg	0.36	0.84	0.13	0.51	1	3.867	0.05
2MWT distance (m)	91.37	48.09	113.73	49.48	1	6.35	0.013
TUG (s)	41.10	53.26	15.16	4.32	1	14.619	0.000
360° turn time L (s)	26.98	94.04	6.25	3.42	1	3.012	0.085
360° turn steps L	13.62	6.75	10.23	4.50	1	10.012	0.002
360° turn time R (s)	14.26	16.05	6.03	2.91	1	15.735	0.000
360° turn steps R	14.73	7.61	10.03	4.09	1	17.229	0.000
	*Yes*	*No*	*Yes*	*No*	*df*	$\chi^{2}$	*p*
Dyskinesia	4	64	28	42	2	23.785	0.000

## Data Availability

The data presented in this study are available on request from the corresponding author. The data are not publicly available due to privacy and ethics issue.
